# Palatal donor site management using tissue adhesives with adjunctive coconut oil in a randomized clinical trial

**DOI:** 10.1038/s41598-025-17071-5

**Published:** 2025-08-26

**Authors:** Melis Yilmaz, Nur Balci, Bestegul Gunay, Hanife Merva Parlak, Ayhan Parmaksiz, Hilal Toygar, H. Gencay Keceli

**Affiliations:** 1 Faculty of Dentistry, Periodontology Department, Istanbul Medipol University, Istanbul, Turkey; 2 Department of Periodontology, Institute of Health Sciences, Istanbul Medipol University, Istanbul, Turkey; 3 Faculty of Dentistry, Periodontology Department, Hacettepe University, Ankara, Turkey; 4 Department of Biostatistics, Istanbul Health & Technology University, Istanbul, Turkey

**Keywords:** Absorbable gelatin sponge, Cyanoacrylates, Coconut oil, Wound healing, Donor site, Tissue grafts, Dental diseases, Oral diseases

## Abstract

Management of the complications occurring in the donor area after epithelialized gingival graft (EGG) harvesting still poses a challenge for clinicians. This study aimed to evaluate the influence of gelatin sponge (GS), cyanoacrylate (CY), and coconut oil (CO) combination in comparison with GS + CY in donor site management. Fifty EGG were harvested from the lateral palate in 50 patients, who were subsequently randomized to receive the GS + CY (control) or GS + CY + CO combination (test). Postoperative pain perception (PP), quantity of analgesics (QA), epithelization level (EL), color match (CM), sensation loss (SL), postoperative discomfort (PD), and delayed bleeding (DB) were evaluated. The GS + CY + CO group showed lower PP scores compared to the GS + CY (*p* < 0.05). The QA intake was similar in both groups. On day 14, the GS + CY + CO group achieved 80% full EL rate, which was higher than the GS + CY group (32%) (*p* = 0.002). CM scores were higher in the GS + CY + CO group at all follow-up periods (*p* < 0.001). Both groups reported similar SL, PD, and DB values (*p* > 0.05). GS + CY + CO combination is more effective in reducing pain compared to GS + CO in donor site management after EGG harvesting. Additionally, GS + CY + CO combination may accelerate epithelialization and improve color match compared to GS + CY alone.

Clinical Trial Registration: ClinicalTrials.gov: NCT06583226.

## Introduction

Epithelialized gingival graft (EGGs) technique is frequently used in soft tissue reconstruction around the teeth, implants or edentulous ridges after obtaining connective tissue grafts via de-epithelialization^1^. EGG has an increasing demand owing to its higher tissue quality, more superficial wound and similar recipient site success rates compared to other harvesting techniques such as trap-door or single-incision^2–4^. Despite the developments in surgical methods and instruments that reduce the depth of the wounds, the secondary healing process after harvesting with EGG still causes significant donor site morbidity. Therefore, clinicians continue to seek ways to reduce these complaints by isolating the wound from the oral environment and/or by accelerating the wound healing with various agents^5,6^. Periodontal dressings, acrylic stents, hemostatic agents, sponges, platelet concentrates, low-level laser therapy, photobiomodulation, cyanoacrylate (CY) tissue adhesives, and hyaluronic acid are the main approaches used in donor site management to accelerate wound healing or to cover the wound surfaces as a barrier^6–10^. Of these, CY, when used with collagen or gelatin sponge (GS), provided promising results compared to other approaches especially in terms of earlier hemostasis with its mechanical barrier effect, improved wound healing, and reducing patient discomfort with its adhesive, hemostatic, and biodegradation properties^6,9^. Parlak et al. reported that CY application after EGG reduced pain and analgesic consumption whereas more positive results could be obtained with additional agents such as hyaluronic acid^9^. Yilmaz et al. stated that the application of the GS + CY combination after EGG treatment helps improve donor site healing by reducing pain perception and secondary bleeding^11^. All these results findings supported the idea that the combination of GS + CY is successful in donor site management, these results are yet to be improved with further agents or methods.

Coconut oil (CO), an edible vegetable oil, contains lauric acid, a precursor of monolaurin. Monolaurin has antimicrobial, anti-inflammatory, bacteriostatic and bactericidal effects through the mechanisms of modulating immune cell proliferation, alteration of bacterial cell walls, penetration and disruption of cell membranes, and inhibition of the enzymes involved in energy production and nutrient transfer^12–14^. In a study, CO showed similar plaque inhibition activity to chlorhexidine mouthwash^15^. CO has also been reported to have an antioxidative effect due to its mineral rich content^16^. In a mice study, topical CO application resulted in accelerated epithelialization and wound healing^16^. However, despite the large number of studies on anti-plaque activity of CO, studies examining its positive effects on wound healing are still necessary.

Due to its barrier and hemostatic properties through antibacterial, antiseptic, antioxidative and immunomodulatory properties, CO may provide additional benefits in donor site management by remaining on the wound surface for an extended duration and exerting sustained biological activity in combination with gelatin sponge and cyanoacrylate, which primarily serve as mechanical barriers. Hypothetically, CO may improve pain and healing outcomes in palatal donor site management when used together with GS + CY. However, to our knowledge, there is no study investigating the efficacy of this combination. Therefore, the aim of this study was to compare the effectiveness of the combination of gelatin sponge, cyanoacrylate, and coconut oil with the combination of gelatin sponge and cyanoacrylate in donor site management following free gingival graft harvesting from the lateral palate.

## Methods

### Study design

**Fig. 1:**
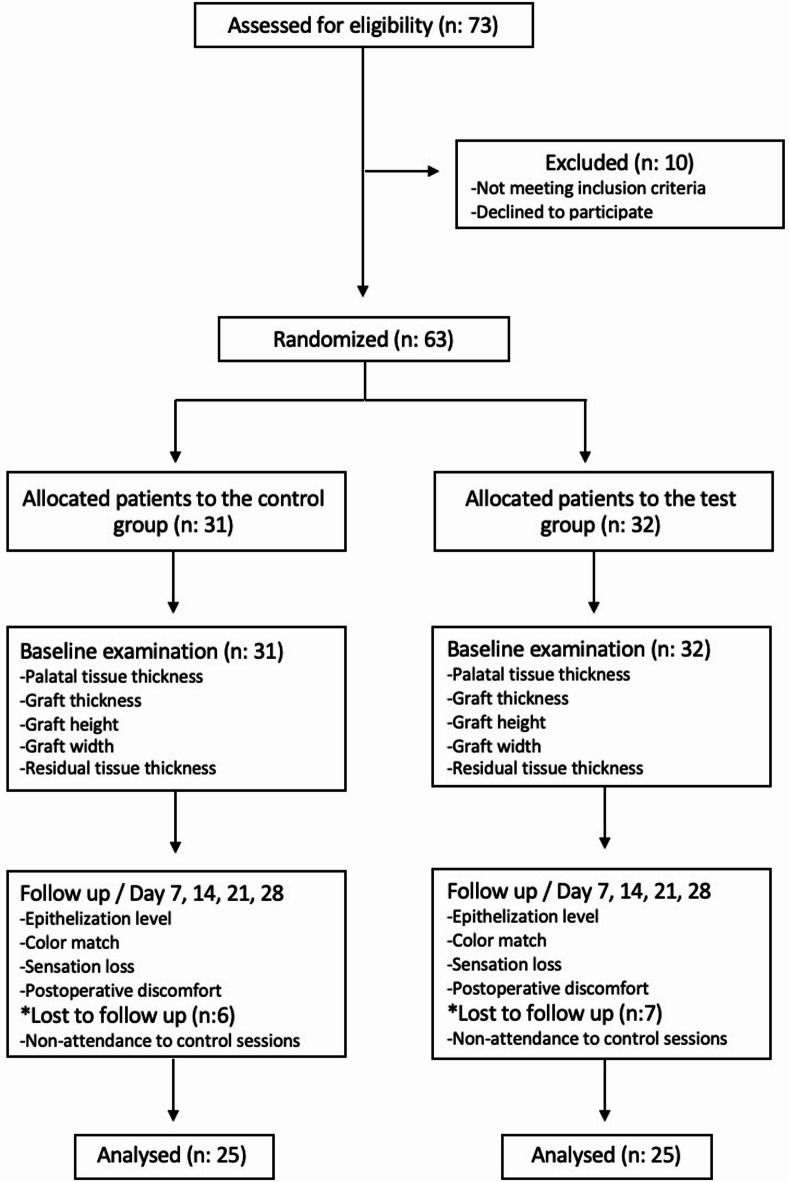
CONSORT (Consolidated Standards of Reporting Trials) flowchart showing the study design.

The study was designed as a prospective, randomized controlled clinical trial including two parallel arms in compliance with the CONSORT statement (Fig. 1), and assessments were performed by masked examiner^17^. The protocol was approved by the Istanbul Medipol University Ethics Committee (10.08.2023/636). The study was conducted by the Helsinki Declaration of 1975, as revised in 2013. The clinical trial was registered at ClinicalTrials.gov (NCT06583226) on 3.09.2024. Informed consent was obtained from all participants included in the study.

### Patient selection

Patients in need of mucogingival surgery were selected from patients referred to the Periodontology Department of Istanbul Medipol University between October 2023 and May 2024 according to the following inclusion criteria: (1) age ≥ 18 years, (2) systemically healthy, and (3) single or multiple recession defects in the lower anterior site and a clinical indication for mucogingival surgery requiring EGG for treatment. The following extrusion criteria were applied: (1) pregnancy and lactation, (2) smoking and alcohol consumption, (3) full-mouth plaque and bleeding scores > 15%, (4) previous palatal harvesting history, and (5) the use of drugs known to affect wound healing.

A total of 73 patients were initially assessed for eligibility. Ten individuals were excluded—either due to not meeting the inclusion criteria or declining to participate—resulting in 63 patients who were enrolled and randomized into the study. Thirty-one patients were allocated to the control group and thirty-two to the test group. During the follow-up period, 6 patients from the control group and 7 patients from the test group were lost to follow-up due to non-attendance at scheduled control visits. Therefore, a total of 50 patients (25 from each group) were included in the final analysis. These details are also illustrated in the flow diagram (Fig. 1). Patients were randomly assigned to receive either GS + CY (control) or GS + CY + CO combination (test), with an allocation ratio of 1:1, using a computer-based randomization program. Allocation was concealed until the time of surgery. ​NB generated the random allocation sequence, enrolled participants, and assigned them to interventions.

### Donor site management and study groups

Epithelialized gingival graft was harvested by the same operator (M.Y) according to the protocol of Zucchelli et al.^3^. After EGG harvesting, bleeding was controlled by compressing wet gauze until it stopped. Then, GS (SURGISPON®, AbGel Pvt. Ltd., India) + CY (PeriAcryl® 90 CE, Glustitch Inc., Canada) or GS + CY + CO (Oneva®, Neva Gıda, Turkey) combinations were applied to the palatal donor site.

Gelatin Sponge + Cyanoacrylate (control) group: GS was used to seal the palatal wound and CY was applied along the wound borders and over the GS. Sling crossed sutures were placed to stabilize the materials using 5 − 0 non-absorbable monofilament.

**Fig. 2:**
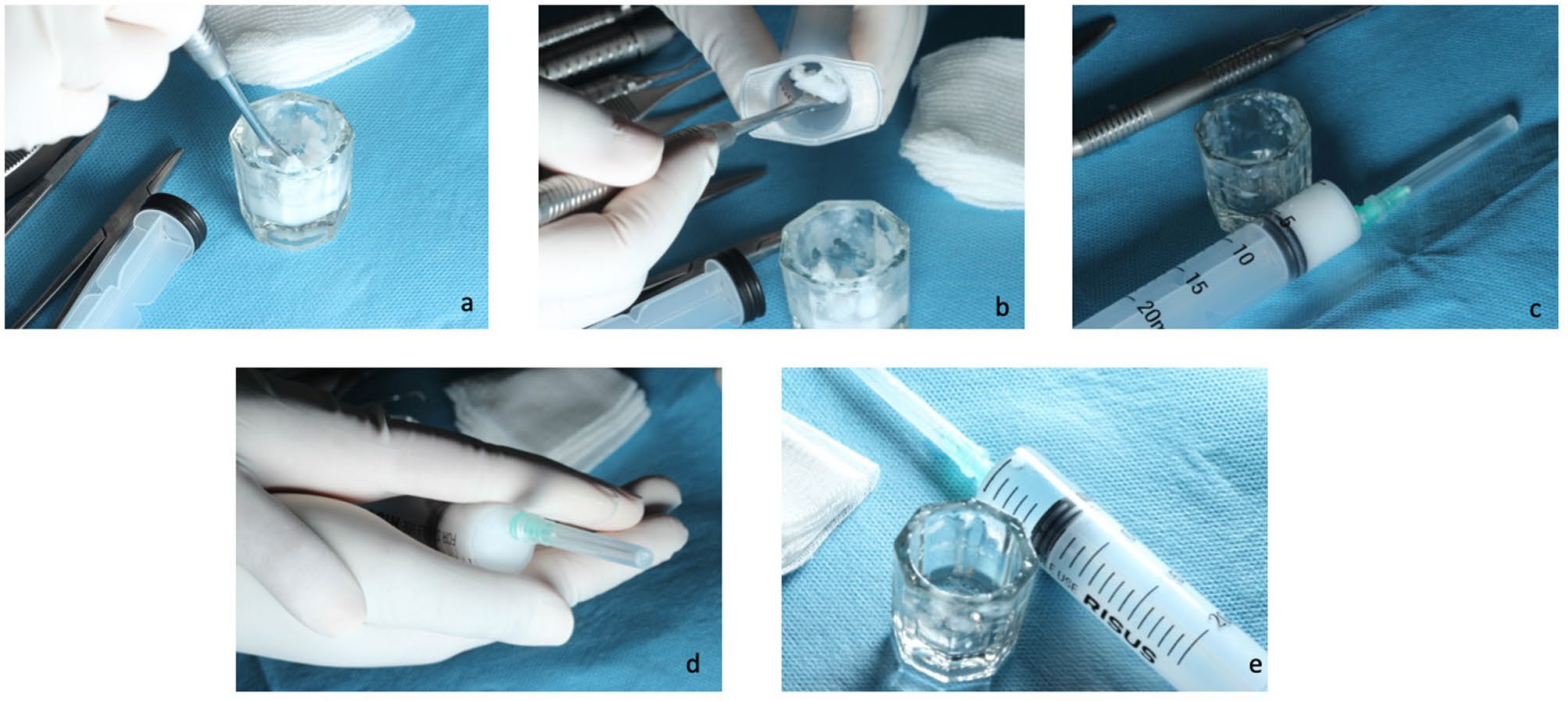
Preparation of coconut oil for clinical application. (a) Collection of coconut oil using a sterile instrument to ensure aseptic handling. (b) Transfer of the oil into a 20 mL sterile syringe under aseptic conditions. (c) View of the 20 mL syringe filled with 5 mL of coconut oil. (d) Warming the syringe by hand to bring the coconut oil to body temperature. E. Coconut oil in liquid form after warming.

**Fig. 3:**
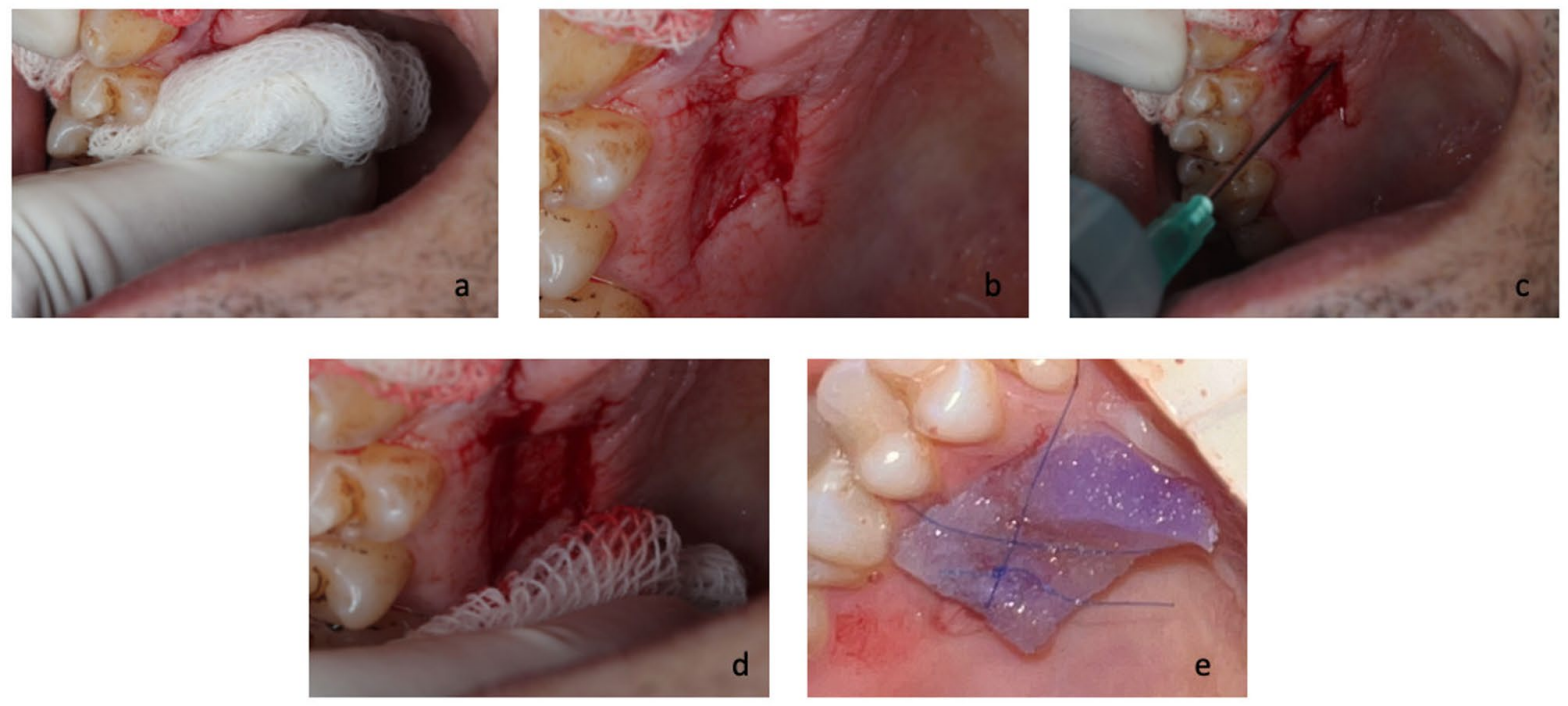
Application of coconut oil to the surgical site. (a) Hemostasis achieved by gentle compression with a moist sterile gauze immediately after EGG harvesting. (b) Palatal donor site following successful hemostasis. (c) Application of 1 mL coconut oil to the donor site using a sterile syringe. (d) Removal of excess oil from the site with sterile gauze to avoid droplet accumulation. (e) Placement of gelatin sponge and tissue adhesive, followed by suturing of the donor site.

Gelatin Sponge + Cyanoacrylate + Coconut Oil (test) group: Once hemostasis was achieved, the site was gently blotted with sterile dry gauze to reduce surface moisture. To ensure aseptic conditions and minimize the risk of contamination, coconut oil was aliquoted under sterile conditions into 20 mL sterile syringes in 5 mL portions. All aliquoting procedures were performed using sterile gloves and instruments. After that, the CO in the sterile syringe was warmed to approximately 37 °C by hand for approximately 1–2 min to obtain a liquid form, and 1mL was then applied to the wound surface using a sterile 20 mL syringe until the area was fully and uniformly covered. Excess oil, if present, was carefully removed from the area beyond the wound margins with sterile gauze to avoid droplet accumulation. Then GS, CY, and suture were performed the same way as the GS + CY group (Figs. 2 and 3)

**Fig. 4:**
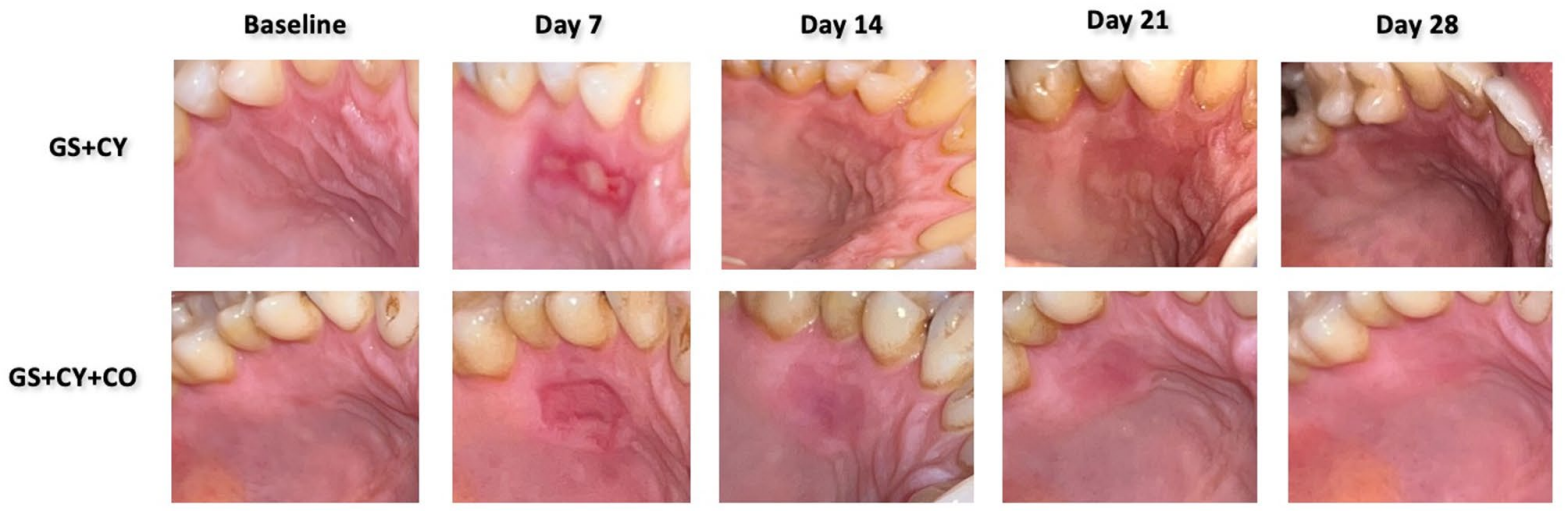
Clinical images of study groups at baseline, and days 7, 14, 21, 28.

Following the surgery, post-surgical instructions were provided to each patient. Analgesic tablets (Flurbiprofen 100 mg) were prescribed for pain control if needed. All patients were instructed to rinse with a mouthwash (0.12% chlorhexidine) twice daily during the first postoperative week. Sutures and cover materials were removed at postoperative 7 days. Patients were followed up on 14, 21, and 28 postoperative days (Fig. 4)

### Sample size

The sample size was calculated using the G*Power based on postoperative pain perception (primary outcome) using data from Keceli et al.^4^. A sample size of 19 patients per study group was necessary to yield 85% power and 5% error level. 25 patients per group were recruited in the trial to allow for 30% dropouts or losses to follow-up.

### Evaluation parameters

The primary outcome of the study was the postoperative pain perception (PP) at the donor site evaluated using a 100-mm visual analog scale (VAS) (0: no pain, 10: severe pain)^6^. Patients were asked to record the presence of delayed bleeding (DB) (yes or no) and the quantity of analgesics consumed (QA) during the first postoperative week.

**Fig. 5:**
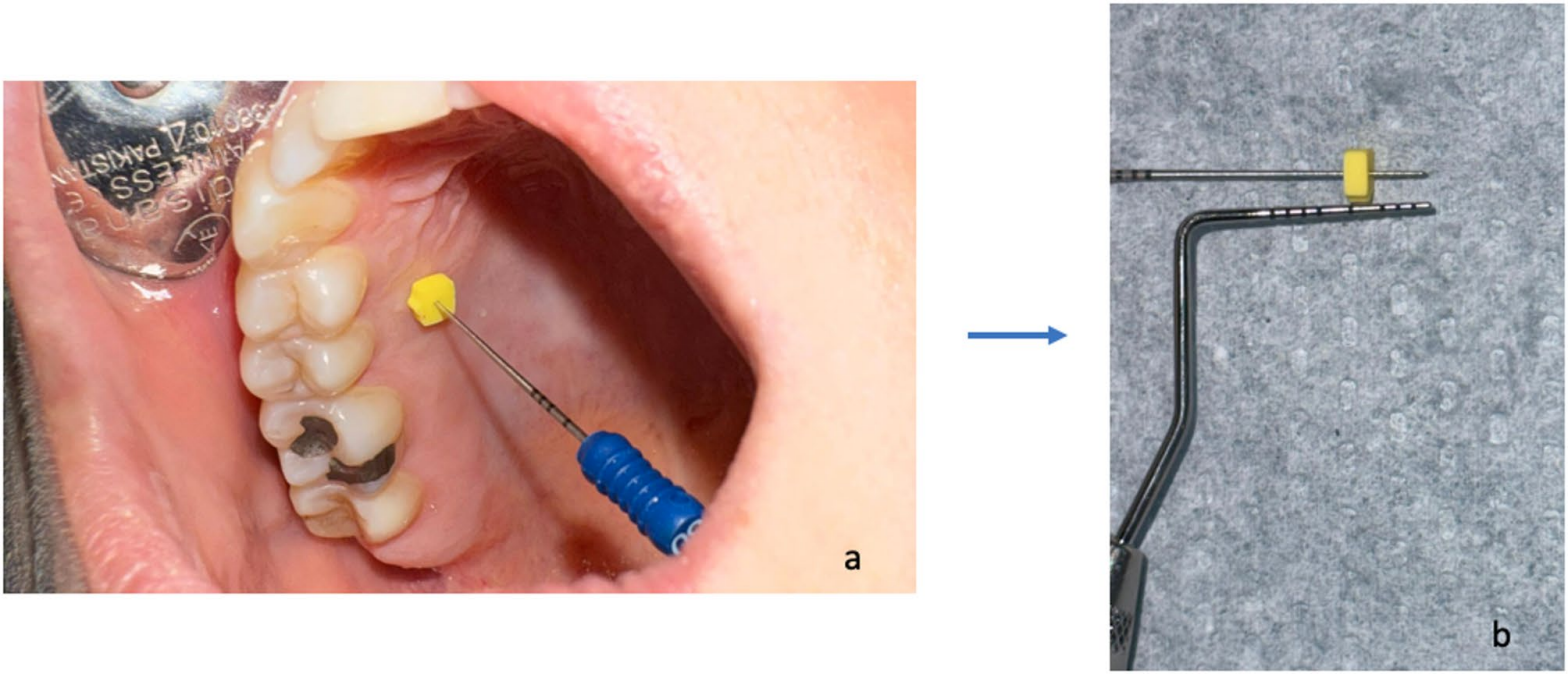
Determination of palatal tissue thickness.

*Intraoperative parameters*: Palatal tissue thickness (PTT) was measured using a 25-endodontic spreader in the mesial, distal, and central parts of the rectangular donor site. The distance between the tip of the spreader and the rubber stopper was recorded with a William’s periodontal probe. (Fig. 5) Graft thickness (GT), graft height (GH), and graft width (GW) were assessed using a periodontal probe (William’s probe, Hu-Friedy, Chicago, IL) after harvesting. Residual tissue thickness (RTT) on the donor site was calculated by subtracting GT from the PTT.

*Postoperative parameters*: Epithelization level (EL) was classified as none, partial, or full based on the visual response to 3% hydrogen peroxide applied to the wound area. The presence and extent of immediate bubble formation were used as indicators of incomplete epithelial coverage^18^. Color match (CM) with the adjacent and contralateral tissues was assessed using VAS (0: no CM, 10: excellent harmony)^19^. Sensation loss (SL) was determined around the donor site with a periodontal probe and patients were asked to rate their SL as none, moderate, or severe (3-point verbal descriptor scale)^20^. Postoperative discomfort (PD) was evaluated by applying a 5-s air blast to the donor site and patients were requested to use VAS (0: no pain, 10: extreme pain) to assess their discomfort^10^. PTT, GT, GH, GW, RTT, EL, and CM measurements were made by the same calibrated author (B.G). EL, CM, SL, and PD were evaluated at 7, 14, 21, and 28 days.

### Statistical analysis

The data were analyzed with SPSS v.27 software (IBM, USA). Quantitative data were expressed as mean ± standard deviation (SD) and qualitative data as numbers and percentages. The significance level was set at 5%. Inter-group comparisons of the qualitative data were made by using independent two samples χ2 test where t-test was used for comparing the quantitative data.

## Results

Fifty patients (25 females, 25 males; mean age: 32.66 ± 7.41 years) completed the study (Fig. 1). No adverse events were reported in both groups. Demographic characteristics of patients, palatal site, and graft size information were presented in Table 1. Age, gender, PTT, GH, GT, and RTT did not show statistically significant inter-group differences (*p* > 0.05). The mean GW in the GS + CY group (9.92 ± 1.15 mm) was significantly higher than in the GS + CY + CO group (9.24 ± 0.88 mm) (*p* = 0.023).

**Table 1:** Demographics, palatal site and graft information.

Parameters	GS + CY (*n* = 25)	GS + CY + CO (*n* = 25)	*p*
Gender†			1.000
Female	13 (52)	13 (52)	
Male	12 (48)	12 (48)	
Age* (years)	33.36 ± 7.49	31.96 ± 7.41	0.510
PTT* (mm)	4.92 ± 0.64	4.64 ± 0.76	0.164
GH* (mm)	5.20 ± 0.76	5.04 ± 0.84	0.485
GW* (mm)	9.92 ± 1.15	9.24 ± 0.88	**0.023**
GT* (mm)	2.48 ± 0.51	2.56 ± 0.51	0.58
RTT* (mm)	2.44 ± 0.77	2.08 ± 0.64	0.078

Age, PTT, GH, GW, GT, RTT, PBT, and SD were shown as mean ± SD while gender values are shown as n (%).

†, χ^2^ test; *, t test.

Statistically significant p values (*p* < 0.05) were indicated in bold.

PTT, palatal tissue thickness; GH, graft height; GW, graft width; GT, graft thickness; RTT, residual tissue thickness; GS, gelatine sponge; CY, cyanoacrylate; CO, coconut oil.

Regarding the primary outcome, the mean PP values were significantly lower in the test group compared to the control group in each of the studied days (*p* < 0.05). The mean QA intake during the first seven days was slightly but not significantly lower in the GS + CY + CO group (2.96 ± 1.14) compared to the GS + CY group (3.20 ± 1.29) (*p* > 0.05) (Table 2).

**Table 2:** Patient reported pain perception (PP) scores and quantity of analgesics (QA).

		GS + CY (*n* = 25)	GS + CY + CO (*n* = 25)	*p*
PP (VAS)†,**	Day 1	5.40 ± 1.12	4.76 ± 0.78	**0.023**
	Day 2	5.16 ± 1.14	4.36 ± 0.64	**0.004**
	Day 3	4.44 ± 1.12	3.76 ± 0.72	**0.014**
	Day 4	3.96 ± 0.89	3.28 ± 0.54	**0.002**
	Day 5	3.60 ± 0.87	2.68 ± 0.63	**< 0.001**
	Day 6	2.76 ± 0.78	2.28 ± 0.68	**0.024**
	Day 7	2.56 ± 0.77	1.88 ± 0.73	**0.002**
QA*		3.20 ± 1.29	2.96 ± 1.14	0.489

PP and QA were shown as mean ± SD.

†, χ2 test; **, Welch t test (day 2 and 5).

*, t-test.

PP, pain perception; QA, quantity of analgesics; GS, gelatine sponge; CY, cyanoacrylate; CO, coconut oil.

Statistically significant p values (*p* < 0.05) were indicated in bold.

In terms of EL, the percentage of patients with none EL was 36% in the control group, while this rate was 20% in the test group, and most of the patients in both groups showed partial EL on day 7 (*p* > 0.05). On day 14, the test group showed 80% full EL rate that was significantly higher than the control group showing 32% (*p* = 0.002). On day 21, the test group showed a slightly but not significantly higher full EL rate (*p* > 0.05). On day 28, all patients in both groups achieved full EL. The mean CM scores were significantly higher in the test group compared to the control group at all follow-up periods (*p* < 0.001). Regarding SL and PD, both groups showed similar scores at all follow-up periods (*p* > 0.05). DB was observed in a few cases in both groups without a statistical difference (*p* > 0.05) (Table 3).

**Table 3:** Epithelialization level (EL), color match (CM), sensation loss (SL), postoperative discomfort (PD), and delayed bleeding (DB) values.

Variable	Day		GS + CY (*n* = 25)	GS + CY + CO (*n* = 25)	*p*
EL^†^ n (%)	Day 7	None	9 (36)	5 (20)	0.226
		Partial	15 (60)	16 (64)	
		Full	1 (4)	4 (16)	
	Day 14	None	4 (16)	1 (4)	**0.002**
		Partial	13 (52)	4 (16)	
		Full	8 (32)	20 (80)	
	Day 21	None	1 (4)	0 (0)	0.171
		Partial	7 (28)	3 (12)	
		Full	17 (68)	22 (88)	
	Day 28	Full	25 (100)	25 (100)	-
CM*,** (Mean ± SD)	Day 7		3.52 ± 0.51	4.80 ± 1.00	**< 0.001**
	Day 14		4.52 ± 0.51	5.80 ± 1.00	**< 0.001**
	Day 21		5.56 ± 0.51	6.88 ± 0.88	**< 0.001**
	Day 28		6.60 ± 0.87	7.80 ± 0.76	**< 0.001**
SL^†^ n (%)	Day 7	None	17 (68)	19 (76)	0.754
		Moderate	8 (32)	6 (24)	
	Day 14	None	25 (100)	25 (100)	-
PD* (Mean ± SD)	Day 7		3.24 ± 1.13	3.12 ± 0.83	0.671
	Day 14		1.48 ± 0.59	1.52 ± 0.51	0.798
	Day 21		1.00 ± 0.00	1.00 ± 0.00	-
	Day 28		1.00 ± 0.00	1.00 ± 0.00	-
DB^†^ n (%)		Yes	2 (8)	1 (4)	1.000
		No	23 (92)	24 (96)	

†, χ2 test; *, t test; **, welch t test (for CM day 7 and 14).

Statistically significant p values (*p* < 0.05) were indicated in bold.

EL, epithelialization level; DB, delayed bleeding; CM, color match; SL, sensation loss; PD, postoperative discomfort; GS, gelatine sponge; CY, cyanoacrylate; CO, coconut oil.

## Discussion

Following EGG harvesting, donor site morbidity including complications of bleeding, pain, and eating difficulties can significantly reduce the patients’ quality of life^4,5^. In the present study, palatal donor sites were managed using the addition of coconut oil to the combination of gelatin sponge and cyanoacrylate, and this was compared with the use of gelatin sponge and cyanoacrylate alone. Both methods improved patient comfort, with a greater effect observed in the group treated with coconut oil.

Coconut oil (CO) is a natural, inexpensive, and easily accessible product derived from coconut, offering various benefits such as antibacterial, anti-inflammatory, and antioxidant properties. Although studies on the effects of CO in periodontal disease primarily focused on its antimicrobial properties, to the best of our knowledge, no study has examined its impact on donor site healing^12,15,21^. During the donor site healing comprised of exudate layer or blood clot formation (initial phase/0–3 days), capillary development and epithelial cell proliferation (second phase/4–10 days), and vascular network formation and epithelial layer maturation into a keratinized structure (third phase/11–42 days), pain and burning sensations peak in the first phase, diminish in the second, and disappear in the third as keratinization completes^22^. Therefore, the present study aimed to evaluate the effects of CO on early wound healing and CO added group showed greater success in reducing pain scores, achieving earlier complete epithelialization, and ensuring better color match compared to the unadded group. Additionally, the pain scores obtained in the GS + CY group were consistent with studies in the literature using this method, while the GS + CY + CO group showed compatibility with the others utilizing different combination treatments^9,11^.

Pain and morbidity at the palatal donor site are affected by multiple dynamics, such as PTT, graft size, and the materials utilized. Among our study groups, there were no significant differences in graft dimensions and RTT scores, except for graft width. However, the pain levels of patients over a 7-day period were significantly lower in the test group compared to the control group. Surgical procedures inevitably induce a trauma-related inflammatory response, leading to pain, edema, and discomfort. However, the anti-inflammatory properties of CO, which have been shown to suppress inflammation-related mediators such as IL-1β, IL-6, and COX-2, may contribute to modulating this response^23^. In line with this, our findings indicate that the use of CO during the wound healing process resulted in a reduction in pain perception. This suggests that CO’s ability to regulate the inflammatory response could be a potential mechanism underlying the observed analgesic effect, contributing to an overall improvement in patient comfort during recovery. Additionally, although GS was used as a protective barrier, CO may have provided an extra layer of protection by forming a thin oil film over the wound surface, shielding it from mechanical trauma. This additional barrier effect could have further contributed to the reduction in pain perception observed in our study.

Studies have reported that CO can be used as a healing agent in burn treatment when compared to silver sulfadiazine^24^. A study evaluating wound healing in diabetic rats reported that rats treated with CO exhibited greater collagen deposition and a more intact epidermal layer compared to those treated with sulfadiazine^25^. Meliala et al. demonstrated that CO plays a role in the wound healing process by inducing the production of MMP-9, PDGF-BB, and TGF-β1 proteins, thereby enhancing proliferation, migration, and angiogenesis^26^. Similarly, in an animal study, CO has been shown to enhance collagen fiber strength and cross-linking, leading to the formation of a more resilient and durable healing tissue. Additionally, it regulates oxidative stress during wound healing, promotes fibroblast proliferation, and stimulates new blood vessel formation^27^. All these mechanisms might have supported the palatal donor site healing process but the exact mechanism should be clarified with further in vivo and clinical studies.

The application of CO during palatoplasty surgery and the use of oil pulling with CO throughout the healing process have also been reported to enhance fibroblast production, accelerate wound healing, and reduce pain complaints^28^. These mechanisms align with our findings, which demonstrated a significant improvement in epithelialization, collagen deposition, and overall wound healing in the CO-treated group. On the 14th day, while complete epithelialization was achieved in 32% of the GS + CY group, this rate was significantly higher at 80% in the CO group. The application of CO, in addition to clot stabilization and wound coverage with the GS + CY combination, effectively accelerated cellular healing, leading to a reduction in epithelialization time. Consequently, this promoted faster healing of the donor site, ensuring a more comfortable recovery process for patients by alleviating pain.

When evaluating donor tissue color matching between groups, the group treated with gelatin sponge, cyanoacrylate, and coconut oil exhibited significantly better color matching than the group treated with gelatin sponge and cyanoacrylate at all time intervals. This finding suggests that, in addition to accelerating wound healing through the previously mentioned mechanisms of proliferation, migration, and angiogenesis, CO may also facilitate a faster restoration of color harmony. However, this should be further confirmed through histological analysis. Furthermore, while CO application demonstrated significant pain reduction, it should be noted that participants were not blinded to the intervention, which may have introduced bias in pain score assessments. Pain scores could have been influenced by patient expectations; thus, future studies should include a placebo arm to control for subjective outcomes. A placebo could have been applied to the GS + CY group to minimize this potential bias. In addition, the preparation and application of CO have not yet been standardized, which represents a limitation of the present study. Finally, the present study excluded smokers and individuals with poor oral hygiene, resulting in a relatively homogeneous cohort. Consequently, the findings may not be generalisable to higher‑risk populations (e.g., smokers, diabetics), and future investigations should recruit more diverse samples to enhance external validity.

## Conclusion

The addition of coconut oil to the combination of gelatin sponge and cyanoacrylate appears to be a successful approach in donor site management following free gingival graft harvesting, resulting in reduced postoperative pain, faster epithelialization, and improved color matching. Although its clinical application is recommended, further research is still needed for a more comprehensive evaluation and clarification of the mechanisms under the palatal tissue healing.

## Data Availability

The datasets generated during and/or analysed during the current study are available from the corresponding author on reasonable request.
